# Genomic transmission analysis of multidrug-resistant Gram-negative bacteria within a newborn unit of a Kenyan tertiary hospital: A four-month prospective colonization study

**DOI:** 10.3389/fcimb.2022.892126

**Published:** 2022-08-25

**Authors:** David Villinger, Tilman G. Schultze, Victor M. Musyoki, Irene Inwani, Jalemba Aluvaala, Lydia Okutoyi, Anna-Henriette Ziegler, Imke Wieters, Christoph Stephan, Beatrice Museve, Volkhard A. J. Kempf, Moses Masika

**Affiliations:** ^1^ Institute of Medical Microbiology and Infection Control, University Hospital Frankfurt, Frankfurt am Main, Hesse, Germany; ^2^ University Center of Infectious Diseases, University Hospital Frankfurt, Frankfurt am Main, Hesse, Germany; ^3^ University Center of Competence for Infection Control, Frankfurt, Hesse, Germany; ^4^ Department of Medical Microbiology, University of Nairobi, Nairobi, Kenya; ^5^ Pediatrics Department, Kenyatta National Hospital, Nairobi, Kenya; ^6^ Quality Health Department, Kenyatta National Hospital, Nairobi, Kenya; ^7^ Center of Internal Medicine/Infectious Diseases Unit, University Hospital Frankfurt, Frankfurt am Main, Hesse, Germany; ^8^ Department of Laboratory Medicine, Kenyatta National Hospital, Nairobi, Kenya

**Keywords:** multidrug resistance, colonization, sub-Sahara, whole genome sequencing, ndm, carbapenemase

## Abstract

**Objective:**

Multidrug-resistant organisms (MDRO), especially carbapenem-resistant organisms (CRO), represent a threat for newborns. This study investigates the colonization prevalence of these pathogens in a newborn unit at a Kenyan tertiary hospital in an integrated approach combining routine microbiology, whole genome sequencing (WGS) and hospital surveillance data.

**Methods:**

The study was performed in the Kenyatta National Hospital (KNH) in 2019 over a four-month period and included 300 mother-baby pairs. A total of 1,097 swabs from newborns (weekly), mothers (once) and the hospital environment were taken. Routine clinical microbiology methods were applied for surveillance. Of the 288 detected MDRO, 160 isolates were analyzed for antimicrobial resistance genes and phylogenetic relatedness using whole genome sequencing (WGS) and bioinformatic analysis.

**Results:**

In maternal vaginal swabs, MDRO detection rate was 15% (n=45/300), including 2% CRO (n=7/300). At admission, MDRO detection rate for neonates was 16% (n=48/300), including 3% CRO (n=8/300) with a threefold increase for MDRO (44%, n=97/218) and a fivefold increase for CRO (14%, n=29/218) until discharge. Among CRO, *K. pneumoniae* harboring *bla*_NDM-1_ (n=20) or *bla*_NDM-5_ (n=16) were most frequent. WGS analysis revealed 20 phylogenetically related transmission clusters (including five CRO clusters). In environmental samples, the MDRO detection rate was 11% (n=18/164), including 2% CRO (n=3/164).

**Conclusion:**

Our study provides a snapshot of MDRO and CRO in a Kenyan NBU. Rather than a large outbreak scenario, data indicate several independent transmission events. The CRO rate among newborns attributed to the spread of NDM-type carbapenemases is worrisome.

## Introduction

Neonatal mortality rates in sub-Sahara Africa, including Kenya, continue to be among the highest worldwide (Gage et al., 2021) and severe newborn infections are accountable for 37% of these deaths (Ahmed et al., 2018). Due to limited medical infrastructure, reduced treatment options and high patient vulnerability (Laxminarayan and Bhutta, 2016), patients in newborn units (NBU) are at high risk for infections with multidrug-resistant organisms (MDRO). A WHO-report of 2017 classifies certain Gram-negative bacteria as critical priority (World Health Organization, 2017) with carbapenem-resistant *Acinetobacter baumannii*, carbapenem-resistant or 3rd generation-cephalosporin resistant *Enterobacteriaceae* and carbapenem-resistant *Pseudomonas aeruginosa* as highest concern. Especially, carbapenem-resistant *A. baumannii* and *Enterobacteriaceae* are prone to cause long-lasting outbreaks in hospital settings (Khalid et al., 2020). Methicillin-resistant *Staphylococcus aureus* (MRSA) are other pathogens often involved in hospital-acquired infections (Schuetz et al., 2021). Surveillance data of MDRO, MRSA and their transmission routes is scarce in low-and middle-income countries (Huynh et al., 2015) but knowledge about it is essential to initiate appropriate infection control measures. In this study, we identified clusters of MDRO and carbapenem-resistant organisms (CRO) at the NBU of the Kenyatta National Hospital (KNH) in Nairobi, Kenya, by an integrated approach combining patient data, routine microbiology results, bacterial genome sequences and infection epidemiology analysis.

## Material and methods

### Study design

This was a prospective study conducted in a newborn unit at KNH between January and April 2019. Rectal swabs from newborns, vaginal swabs from mothers and environmental samples from surfaces and medical equipment (on study days d55-57 and d89) were taken over a period of four months. These samples were analyzed for the presence of MDRO (Department of Medical Microbiology, University of Nairobi (UoN) and the Department of Laboratory Medicine, KNH). For a subset of MDRO, whole-genome-sequencing was performed, and phylogenetic relatedness of the bacterial isolates was assessed (Institute of Medical Microbiology and Infection Control, University Hospital Frankfurt am Main, Germany). Clusters were analyzed by integrated metadata analysis (sampling, location within the hospital).

### Study participants

In this study, 300 mother-newborn pairs over a period of 110 days were included. Inclusion criteria were (i) admission to the NBU and (ii) given informed consent. Exclusion criteria was any given medical or ethical contradiction to rectal swabs of newborns or vaginal swabs of mothers. Ethical approval was given by KNH – UoN Ethics & Research Committee (KNH/UoN-ERC: P208/04/2018; University of Nairobi, Kenya, College of Health Sciences, July 11th, 2018) and by the Ethics Committee of the Medical Faculty Goethe University Frankfurt am Main, Germany (FKZ 01KA1772; 15/05/2018).

### Study site

At KNH NBU, 200 to 300 newborns per month receive medical care. NBU is organized into nine sub-units. At admission, newborns are examined in the admission room. Medical care is provided in newborn intensive care units (NICU1, NICU2, NICU3), in nurseries (nursery B1, nursery B2, nursery B3) or in an isolation room. Nursery D is reserved for newborns with improved health condition. The delivery ward is separated from NBU by two floors. Mothers stay in different post-natal wards and visit the NBU every three hours to (breast-)feed their babies.

### Sample collection

Vaginal swabs (MK Plast, New Delhi, India) were collected from mothers once at the day their newborns were admitted (according to the ethics proposal KNH/UoN-ERC: P208/04/2018). Rectal swabs of newborns were taken at the day of admission, weekly during their stay at NBU and at discharge from the NBU. Due to ethical reasons, no sample was taken from deceased newborns. Environmental samples were categorized as (i) medical devices (e.g., ventilators, ultrasound transducer), (ii) near patient (e.g., cots, incubators), (iii) far from patient (e.g., desks, computer equipment) or (iv) unclean areas (e.g., surfaces, sinks), and were taken on d55-57 and d89.

### Routine microbiology testing

Bacterial cultures were incubated for 24 hours at 35-37°C on selective chromogenic ESBL agar (CHROMagar, Mast, Paris, France). Identification (ID) and antimicrobial susceptibility testing (AST) was done via VITEK-2 (bioMérieux SA, Marcy-l’Étoile, France) using GN83 and P580 cards and imipenem E-test strips (Liofilchem, Roseto degli Abruzzi, Italy) according to Clinical Laboratory Standards Institute (CLSI) guidelines (Clinical and Laboratory Standards Institute (CLSI), 2019). Each ID and AST included a purity control on Columbia blood agar. All MDRO isolates were stored at -80°C in CRYOBANK™ medium (Mast).

### Sequencing

Due to the agreements of the ethics proposal (KNH/UoN-ERC: P208/04/2018), 160 bacterial isolates (including 51/63 CRO) were selected from 288 detected MDRO for whole genome sequencing (WGS) prioritized by the following criteria: (i) carbapenem-resistant phenotype, (ii) culturable bacterial status upon arrival in Germany and (iii) likeliness of transmission onto or among newborns. Isolates were shipped on dry ice in CRYOBANK medium to Frankfurt am Main, Germany and were phenotypically re-assessed upon arrival using routine microbiology methods. Identification and AST were confirmed using Vitek-2, ID MALDI-ToF MS (bioMérieux SA, Nürtingen, Germany) according to European Committee on Antimicrobial Susceptibility Testing (EUCAST) guidelines version 8.0 (accessible via https://www.eucast.org/clinical_breakpoints/). Lateral flow assays (Hardy, Santa Maria, USA) were used to detect the following carbapenemases: NDM, KPC, OXA-48, VIM and IMP. All laboratory testing was performed under strict quality control criteria (laboratory accreditation according to ISO 15189:2011 standards) at the Institute for Medical Microbiology and Infection Control, University Hospital Frankfurt am Main, Germany. Isolates with inconsistent AST, unclear documented origin and copy strains (meaning that the same pathogen was detected in the same newborn multiple times) were excluded from further analysis.

DNA of cultured bacteria was extracted using DNeasy UltraClean 96 Kit (Qiagen, Venlo, Netherlands). Library preparation and sequencing was performed by a commercial service provider (Novogene, Cambridge, UK) using Illumina chemistry. Sequencing was carried out on a NovaSeq 6000 flow cell using a paired-end sequencing strategy of 2x150 bp. Details for *in silico*-sequence analysis is described in the **Supplementary Information**.

### Software/Statistics

Research Electronic Data Capture software (REDCap, Vanderbilt University, Nashville, USA) was used to capture and store sample metadata (e.g., sample type, sampling date and time, location) and resistance phenotype (including ID, AST and subcultures). Confidence intervals (CI) were calculated using Newcombe-Wilson (Newcombe, 1998) method and the Relative Risk (RR) was determined using the Armitage -Berry Methods (Armitage et al., 2008).

## Results

### Sample collection and phenotypic determination of antimicrobial resistance

A total of 300 mother-newborn pairs were included in this study. The median age of the mothers was 27 years, and the range of newborn gestation age was between 25 weeks+6 days to 42 weeks+0 days. These newborns were admitted to the NBU at the day of delivery (or immediately after referral from other hospitals).

**Figure 1:**
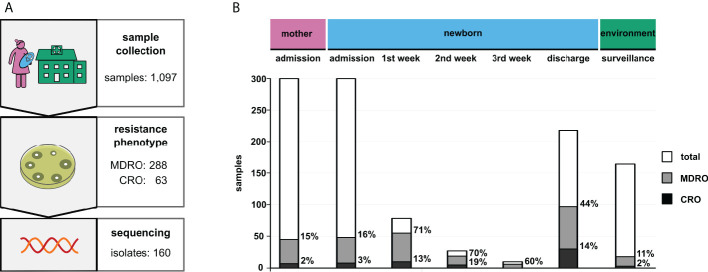
Results of the surveillance study. **(A)** Study design including the respective sample numbers (MDRO, multidrug-resistant organisms; CRO, carbapenem-resistant organisms). **(B)** Total prevalence of the identified bacteria grouped by sample type. “Admission” and “discharge” refer to the particular hospital stays of patients. Column sizes indicate absolute numbers, while percentages of MDRO and CRO are given next to the respective columns.

In total, 1,097 swabs were obtained, including 164 environmental samples (**Figure 1A**). Among the 288 detected MDRO, the most frequent species was *K. pneumoniae* (n=155) followed by *E. coli* (n=83) and *A. baumannii* (n=7). Multidrug-resistant *P. aeruginosa* isolates were not detected in any sample. Furthermore, 63 bacterial isolates were identified as CRO (*K. pneumoniae*: n=35; *E. coli*: n=13; *A. baumannii*: n=5).

At admission to NBU (after delivery or after referral from another hospital), MDRO were detected from 16% of newborns (n=48/300; 16%; CI 12-21%), including 3% CRO (n=8/300; 3%; CI 1-5%). Among mothers, a 15% MDRO rate (n=45/300; 15%; CI 11-19%), including 2% CRO (n=7/300; 2%; CI 1-5%) was observed. The rates for mothers and newborns at admission were similar (MDRO: RR 0.94; CI 0.65-1.36; CRO: RR 0.88; CI 0.32-2.38). For newborns, the rate of MDRO increased from admission to discharge from 16% to 44% (n=97/218; 44%; CI 38-51%), and for CRO from 3% to 14% (n=29/218; 14%; CI 9-18%) indicating that 49 newborns became colonized with MDRO and of these, 21 newborns with CRO. This represents a three-fold increase of MDRO and a five-fold increase of CRO (MDRO: RR 2.78; CI 2.06-3.75; CRO: RR 4.99 CI 2.33-10.70). The highest increase was observed in the first week after admission to NBU (see **Figure 1B).**

MDRO and CRO isolates were obtained from medical devices (n=9), unclean areas (n=6) and near patient areas (n=3). Rates in NBU-environmental samples were 11% (n=18/164; 11%; CI 7-17%) for MDRO and 2% (n=3/164; 2%; CI 1-5%) for CRO.

### Genomic characterization and phylogenetic analysis of MDRO and CRO

Of all sequenced *Enterobacteriaceae*, 89% (n=137/154) harbored an extended spectrum β‐lactamase (ESBL) type *bla*_CTX-M-15_. For 42/51 sequenced CRO, a *bla*_NDM_-type carbapenemase gene was identified. These were distributed among *K. pneumoniae* (n=25; *bla*_NDM-1_: n=14, *bla*_NDM-5_: n=10, *bla*_NDM-7_: n=1), *E. coli* (n=11; *bla*_NDM-5_: n=6, *bla*_NDM-7_: n=5) and other *Enterobacteriaceae* (n=6; all harboring *bla*_NDM-1_). For all four *A. baumannii* isolates, a *bla*_OXA‐23_ carbapenemase was detected; one of those additionally harbored *bla*_OXA‐66_, two others *bla*_OXA‐69_ and one *bla*_OXA‐365_. Other detected carbapenemases include *bla*_OXA‐232_ and *bla*_OXA‐181_ (each found in one *K. pneumoniae* isolate, respectively). Detailed information regarding sequence type and antimicrobial resistance genes is given in **Supplementary Table 1**.

**Figure 2:**
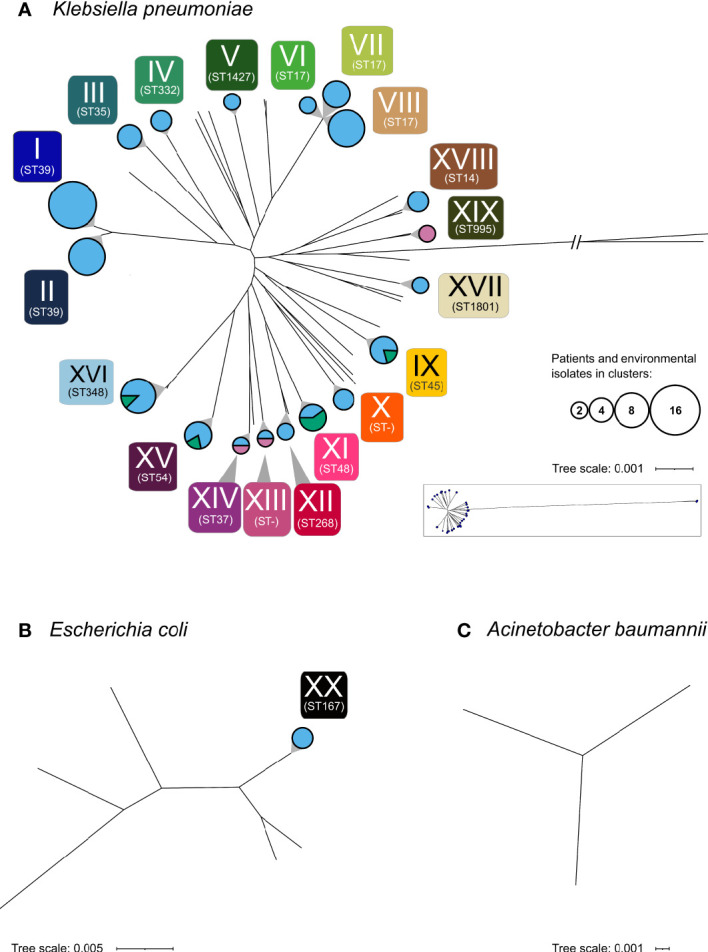
Phylogenetic analysis of the 160 sequenced isolates. Phylogenetic analysis revealed 20 clusters (I-XX) depicted as circles. **(A)** *K. pneumoniae*, **(B)** *E. coli*, **(C)** *A. baumannii.* Circle areas represent the number of patients and environmental samples forming the cluster, while the circle color indicates the respective sample types (rose, maternal; light blue, neonatal; green, environmental). Multilocus sequence types (ST) of clusters are shown below each cluster. Non-typable sequence types are designated as “ST-”. Sequence types of all particular isolates are given in **Supplementary Table 1**.

A phylogenetic analysis of the 160 selected isolates was carried out. Copy strains (n=17) were excluded once confirmed by sequence analysis. Results revealed 20 clusters of closely related isolates, including five CRO clusters. Of these, 19 were formed by *K. pneumoniae* (CRO clusters: n=4) and one cluster was formed by *E. coli* (see **Figure 2** and **Supplementary Table 2**).

Among these clusters, cluster I and II as well as cluster VI, VII and VIII consist of isolates of the same sequence type. The median difference between isolates of cluster VI and VII is 132 SNVs (min: 130; max: 133), of cluster VI and VIII 182 SNVs (min: 175; max: 184) and of cluster VII and VIII 191 SNVs (min: 183; max: 193), respectively. Similarly, cluster I and II both belong to ST39, with a median difference between the isolates of 2,380 SNVs (min: 2,380, max: 2,384). These results demonstrate that these clusters VI, VII and VIII are distinguishable within ST 17 and clusters I and II within ST 39.

Cluster I, which consist of *K. pneumoniae* ST39 with *bla*_CTX-M-15_, represents the largest cluster (n=15). The isolates spanned the complete investigated period (d14 until d106) and were obtained from 15 neonates in seven of the nine NBU-subunits (except isolation room and NICU3).

**Figure 3:**
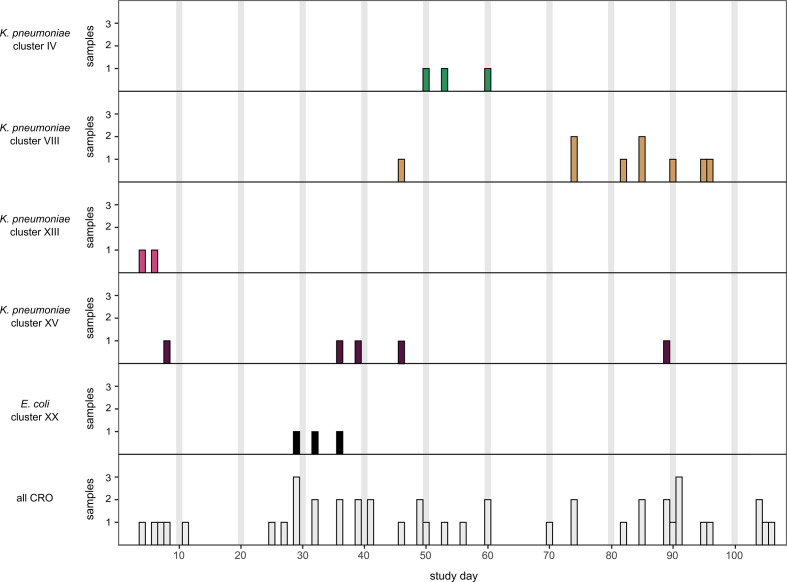
Surveillance timeline of CRO over 110 study days. From 51 detected CRO, seven copy strains were excluded resulting in 44 unique isolates. In separate rows, the *K. pneumoniae clusters* IV, VIII, XIII, XV and the *E. coli* cluster XX are displayed.

Cluster VIII, formed by *K. pneumoniae* isolates of ST17 carrying *bla*_NDM-5_, was the largest CRO cluster (**Figure 3**). The respective isolates derived from nine different newborns and were obtained from three different subunits (**Supplementary Figure 1**). The initial isolate was sampled on Nursery B3 on d46. Six isolates were detected in Nursery D (on d74 (n=2), d82, d85 (n=2) and d96) and two in NICU2 on d90 and d95, respectively.

Clusters indicating transmissions among mothers and newborns were rarely found (only cluster XIII and XIV). Only in three cases, bacteria of the same species (*K. pneumoniae* with MDRO status) were detected in mothers and their respective newborns but none of these bacteria were phylogenetically closely related (pairwise SNP distance: 16,780, 16,583 and >133k SNPs, respectively) excluding vertical transmission from mother to child. Clusters consisting of isolates obtained from the NBU-environment and among newborn samples (cluster IX) as well as clusters consisting exclusively of samples from mothers (cluster XIX) were detected. Besides the already mentioned ST39 (n=26; cluster I: n=15, cluster II: n=9; no cluster: n=2), ST17 (n=17; including the NDM-5-positive cluster VIII: n=9) and ST348 (n=13; no carbapenemase detected) were the most frequently found *K. pneumoniae* sequence types.

Plasmid MLST analysis and genomic assessment of those regions flanking carbapenemase genes indicate that transmission of bacteria rather than plasmid hospitalism is the dominant mechanism for the spread of carbapenemases and the occurrence of CRO (see **Supplementary Figure 2, Supplementary Figure 3**).

## Discussion

This report focusses on MDRO colonization prevalence among newborns in a tertiary hospital in Kenya with a special emphasis on carbapenem resistance. Data revealed a five-fold increase of CRO from 3% at admission to 14% at discharge underlining the need for appropriate infection control actions. Genomic analysis revealed 20 MDRO clusters and, in particular, five heterogeneous CRO clusters (clusters: VIII: n=9 isolates; XV: n=5; IV: n=3; XX: n=3; XIII: n=2; see **Supplementary Table 2**) within the relatively short study period. These results indicate not one ongoing outbreak scenario but several individual transmissions and emphasize a need for multiform counteractions which are not easy to implement in clinical routine patient care.

Data on MDRO colonization prevalence among NBUs in low- and middle-income countries is limited (Huynh et al., 2015) and studies are often focussed on clinical infections while the colonisation status (a prerequisite for infection) is not reported. The most prevalent sepsis-causing pathogens in NBUs in sub-Saharan Africa are *S. aureus*, *Klebsiella spp.* and *E. coli* (Okomo et al., 2019). In our study, screening did not detect any MRSA (data not shown). While similar to our findings, a previous study from two hospitals in Nairobi, Kenya (Omuse et al., 2015) reported only a low MRSA prevalence (3.7%), in our study only vaginal and rectal swabs were included, which are known to be of limited sensitivity for MRSA detection (Bitterman et al., 2010). The absence of multidrug-resistant *P. aeruginosa* in other sub-Saharan NBUs (Ghana) is also consistent with our results (Labi et al., 2020).

An earlier Kenyan study reported ESBL colonization rates of 10% at admission to NBU with an incidence of acquisition of 21.4% per day resulting in more than half of the neonates to be colonized with ESBL within the first three days upon admission (Kagia et al., 2019). In Ghana (Labi et al., 2020), 75% of the *Klebsiella spp.* from NBUs were ESBL positive and the carriage rate of carbapenemase-producing *Klebsiella spp.* was 8%. This shows that, the MDRO colonization rate among newborns in this study is high but within the reported range from sub-Saharan Africa (Kagia et al., 2019; Labi et al., 2020). In contrast, a study from a German NBUs disclosed Denkel et al., 2014 (Rettedal et al., 2015) found 2.9% of mothers to be colonized with ESBL.

The high rate of CRO-colonized newborns at discharge (14%) is alarming but in range with results from other sub-Saharan studies (8-9% in South Africa (Ballot et al., 2019) and Ghana (Labi et al., 2020)). Consistently, when looking at neonatal sepsis, an increase of CRO from about 3% (2013) to 9% (2015) was detected in South Africa due to NDM-producing *K. pneumoniae* (Ballot et al., 2019) but the underlying NDM-subtype remained unreported. Also, high CRO rates (e.g. 24% carbapenem resistance among *K. pneumoniae*) in clinical isolates at KNH have been described earlier (Wangai et al., 2019). These reports indicate that CRO represent a significant threat for patients and, in particular, for newborns in Kenya and other sub-Saharan African countries.

*K. pneumoniae* NDM-1 was initially detected in Nairobi in the year 2007 (Poirel et al., 2011). Among more than 200 studies from 2010 to 2019 analysing the prevalence of NDM in Africa, NDM-1 was dominating by far (93%) with much lower rates for NDM-5 (4%) and NDM-7 (2%) (Safavi et al., 2020). *Enterobacteriaceae* from Kenyan hospitals were reported earlier to harbor NDM-1 and NDM-5 and for *A. baumannii* OXA-23 was found to be most prevalent (Musila et al., 2021). This carbapenemase pattern is widely reflecting the distribution of CRO characterized in our study.

MDRO outbreaks in NBUs are frequently reported and whole genome sequence analysis has proven a powerful tool for outbreak analysis (Mammina et al., 2007; Dramowski et al., 2017; Johnson and Quach, 2017; Brinkac et al., 2019; Okomo et al., 2020). Usually, problems in basic hygiene and increased exposure to medical procedures are significantly associated with MDRO infections (Haller et al., 2015). Such basic hygiene problems (possibly originating from medical staff or mothers, or visitors) are reflected by the high rate of MDRO/CRO detections from environmental samples (MDRO: n=18/164; CRO: n=3/164) and are difficult to overcome.

Shortcomings in basic hygiene contributed, e.g., to a *K. pneumoniae* ST39 outbreak in Gambia (Okomo et al., 2020) and this sequence type was also the prevalent among MDRO isolates (cluster I and II; n=25) in our study. In KNH, we found 20 different clusters suggesting several independently occurring transmission events over all NBU subunits with MDRO isolates from mothers and environmental samples (see **Supplementary Figure 1**). Unfortunately, exact transmission routes could not be reconstructed as this topic was not part of the initial study protocol. Clearly, the high MDRO entry by mothers (15% MDRO, 2% CRO) and newborns (16% MDRO, 3% CRO) at admission is a challenge for any infection control team.

To mitigate against this threat to newborns, staff at the KNH NBU have implemented multiple infection control measures (e.g., infection control team with weekly ward rounds, antibiotic stewardship team with daily consultations) and supported the analysis of the MDRO/CRO prevalence and transmission events strongly. Also, transmission events were clearly detected at the NBU, the successful work of the team is reflected by the fact that 56% of the newborns were not colonized with MDRO at discharge.

The herein described MDRO and CRO prevalence at the NBUs of KNH is worrisome and needs further attention (i) to clarify transmission routes and (ii) to implement further infection control measures. Generally, the limited MDRO surveillance data from sub-Saharan Africa indicate an increase of CRO infections in recent years but studies analysing colonization rather than infections are scarce (Okomo et al., 2019). It must be assumed that the extend of antibiotic resistance in Kenya is underestimated.

## Data availability statement

Sequence data generated in this study was deposited in the NCBI Sequence Read Archive (SRA) under BioProject accession PRJNA804332.

## Ethics statement

Ethical approval was given by KNH – UoN Ethics & Research Committee (KNH/UoN-ERC: P208/04/2018; University of Nairobi, Kenya, College of Health Sciences, July 11th, 2018) and by the Ethics Committee of the Medical Faculty Goethe University Frankfurt am Main, Germany (FKZ 01KA1772; 15/05/2018).

## Authors contributions

General conceptualization: DV, VK, II, IW, and LO. Concept design and project management: DV and MM. Data collection and bacteriology: DV, MM, A-HZ, BM, VM, JA, IW, and LO. Data analysis non-WGS: DV, VM, A-HZ, TS, and VK. Data analysis WGS and Figure design: TS and DV. Writing of the manuscript DV, TS, VK, MM, and CS, II. All authors contributed to the article and approved the submitted version.

## Funding

The authors have no competing interests to disclose. Funding for this study was provided by DLR (Deutsches Zentrum für Luft- und Raumfahrt) in cooperation with German Federal Ministry of Education and Research (BMBF; grant number 01KA1772) and partially by the LOEWE Center DRUID (Novel Drug Targets against Poverty-Related and Neglected Tropical Infectious Diseases). Findings and conclusions of this study do not necessarily represent views of the University.

## Acknowledgments

We thank all laboratory and clinical staff at KNH and UHF involved in the study, in particular G. Revathi (Aga Khan University Hospital) and B. Maugo, M. Alacoque, S. Kinara and C. Onsinyo (all KNH).

## Conflict of interest

The authors declare that the research was conducted in the absence of any commercial or financial relationships that could be construed as a potential conflict of interest.

## Publisher’s note

All claims expressed in this article are solely those of the authors and do not necessarily represent those of their affiliated organizations, or those of the publisher, the editors and the reviewers. Any product that may be evaluated in this article, or claim that may be made by its manufacturer, is not guaranteed or endorsed by the publisher.
