# Supplementary material for: Genomic transmission analysis of multidrug-resistant Gram-negative bacteria within a newborn unit of a Kenyan tertiary hospital: A four-month prospective colonization study
Source: Front Cell Infect Microbiol. 2022 Aug 25;12:892126. doi: 10.3389/fcimb.2022.892126 (PMC9452910; doi:10.3389/fcimb.2022.892126)
Supplement: Supplementary file 1 [file DataSheet_1.pdf]

Supplementary Material

1 Material & Methods

1.1 Sequence processing and *de novo* assembly

A quality assessment was performed on all sequencing files using fastqc v.0.11.8 (Andrews S, Lindenbaum P, Howard B) and remaining partial adapter sequences were removed with cutadapt (Martin, 2011) version 2.5. *De novo* assembly was carried out using Unicycler v0.4.8-beta as an optimizer for SPAdes (Prjibelski et al., 2020). Assembly statistics were obtained using a script under R(R Core Team) version 3.4.4 utilizing the package seqinr (Charif and Lobry, 2007). For study inclusion, (i) total assembled length of each particular bacterial isolate had to be consistent with the expectations from literature (*Klebsiella spp.*: 5 to 7 Mb; *A. baumannii* 3.5 to 4.5 Mb; *Citrobacter spp*., *Enterobacter cloacae* and *E. coli*: 4 to 6 Mb), (ii) the smallest amount of contigs with a cumulative size equal or bigger than half the genome size (L50) had to be lower than 20 and (iii) the total amount of contigs larger than 1kb had to be below 250.

1.2 Multilocus sequence typing, phylogenetic analysis and plasmid typing

The seven-gene multilocus sequence type (MLST) for each isolate was determined by using MLST (Seemann) version 2.18.0 and PubMLST (https://pubmlst.org/) (Jolley and Maiden, 2010) database as reference. For annotation, Prokka (Seemann, 2014) version 1.14.6 was utilized. Pan-genome analysis was carried out by using for Roary (Page et al., 2015) version 3.13.0 with the default parameters. Nucleotide alignments of the core genome were used to construct a maximum likelihood tree with fasttree (Price et al., 2010) v2.1.10 using a general time reversible model. To access support of the nodes, 100 random bootstrap replicates were performed. Snp-dists (Seemann) v0.7.0 was used to calculate a pairwise distance matrix of single nucleotide differences in the core genome for all isolates of a species. As a mean to visualize similarity of isolates on a whole genome level, gene presence-absence plots were generated utilizing a community-contributed python script (accessible via https://raw.githubusercontent.com/sanger-pathogens/Roary/master/contrib/roary_plots/roary_plots.py). Plasmid typing was carried out using Plasmid MLST (Jolley and Maiden, 2010) (database version: June 22nd, 2022). For analysis of flanking genes, read files were mapped against the respective reference using bowtie2 (Langmead and Salzberg, 2012). Alignments were assessed using CLC Genomics workbench version 22.0.

1.3 *In-silico* analysis of antimicrobial resistance genes

Identification of bacterial antibiotic resistance genes was carried out by screening of all assemblies against CARD (Jia et al., 2017) using abricate (Seemann) version 0.9.8. For visualization, fractions of the matched sequence ("hits") were summarized and imported into R using the readR (Wickham et al., 2018) package. In cases where several hits were found for one resistance gene, only the best hit was considered.

2 Supplementary figures

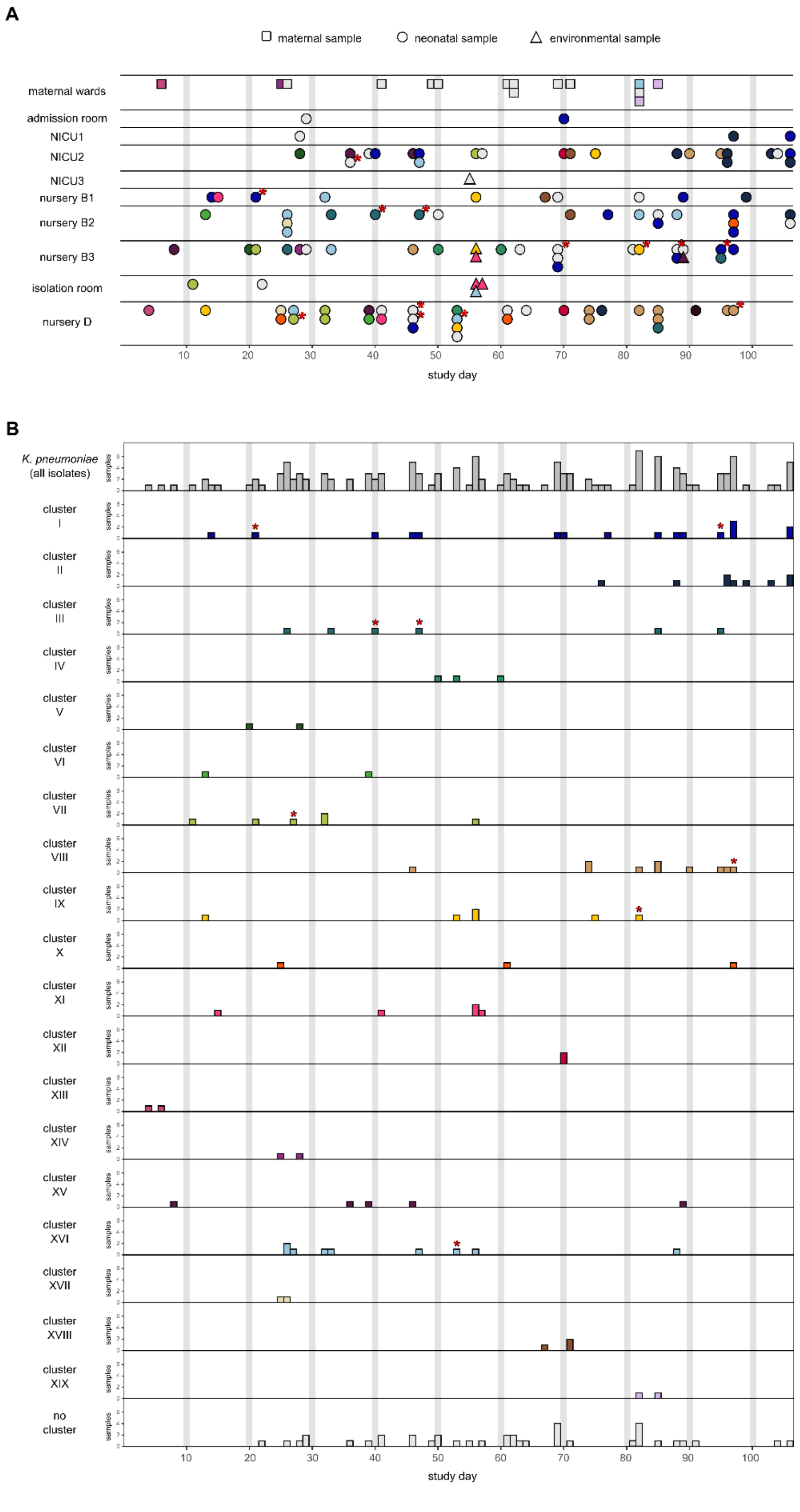

Supplementary Figure 1: Surveillance timeline (160 sequenced isolates) over 110 study days. (A) Analyzed isolates are grouped by wards in which the respective bacterial isolate was detected (maternal, neonatal, environmental samples). Colors indicate the respective cluster affiliation. (B) Depiction of the timeline for individual clusters. Copy strains are marked by red asterisk.

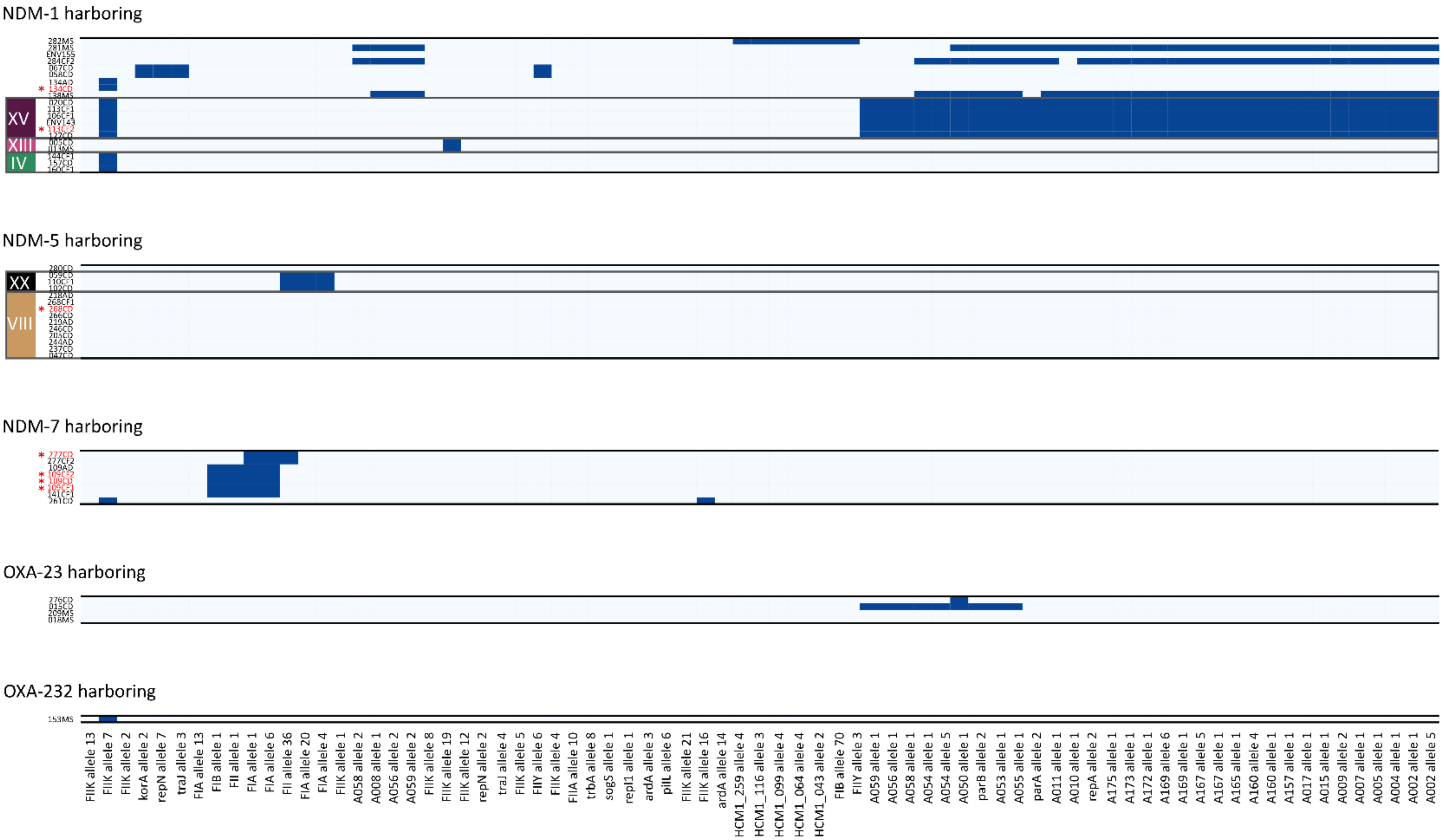

Supplementary Figure 2: **Plasmid MLST profiles of isolates with a detected carbapenemase gene.** Exact matches of allele types are indicated by blue fields. Each colored box represents a cluster (derived from core genome phylogeny). Copy strains are marked by red asterisks.

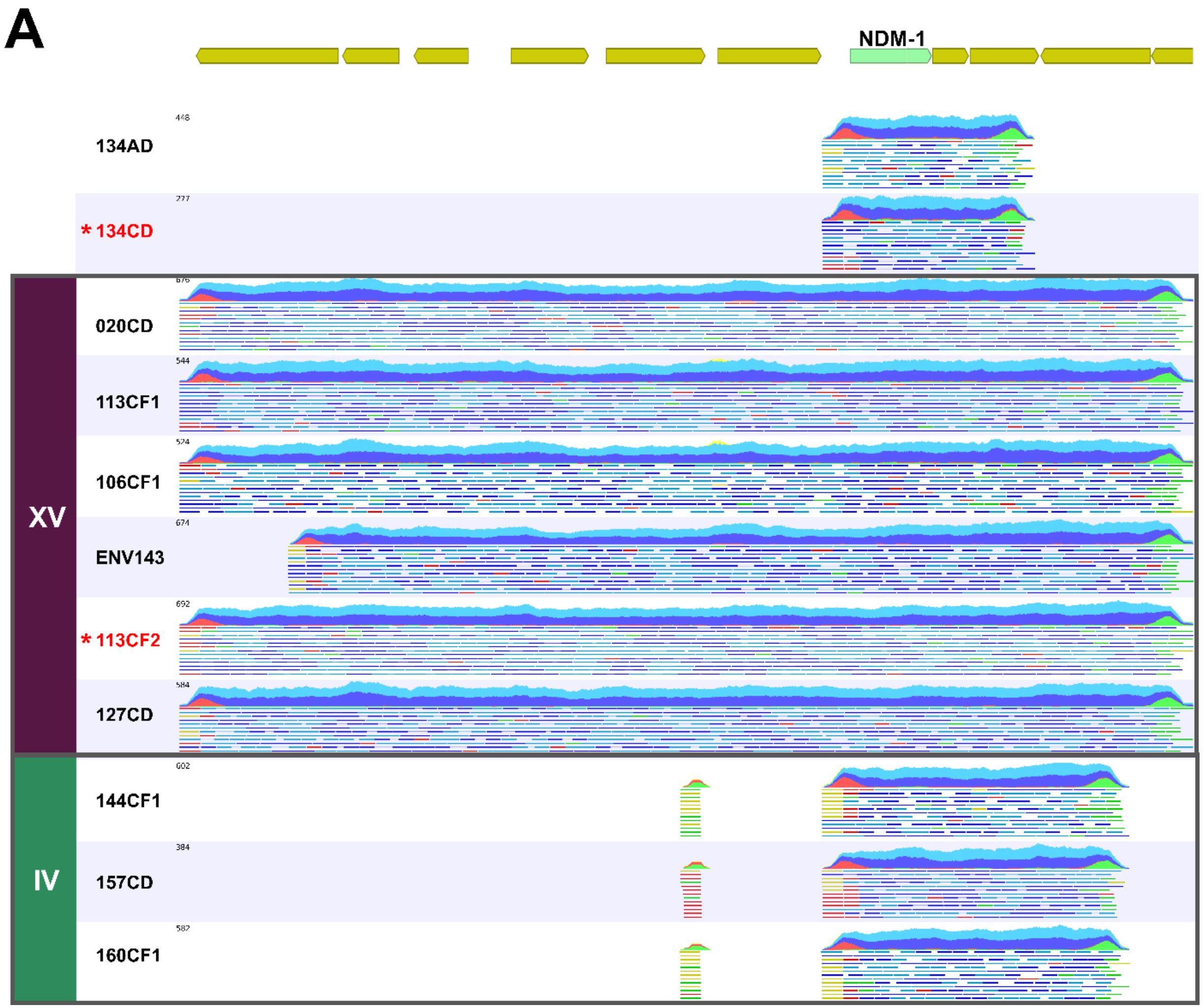

Supplementary Figure 3A: **Genes flanking *bla*NDM-1.** To access similarity of flanking regions, all *bla*NDM-1-positive isolates for which FIIK allele 7 was found were assessed using a read mapping-based approach. Read files of respective isolates were mapped against the plasmid contig of isolate #020CD. Each colored box represents a cluster (derived from core genome phylogeny). Copy strains are marked by red asterisks. Results indicate differing flanking regions for isolates from cluster XV, cluster IV and those from newborn 134 (#134AD and #134CD).

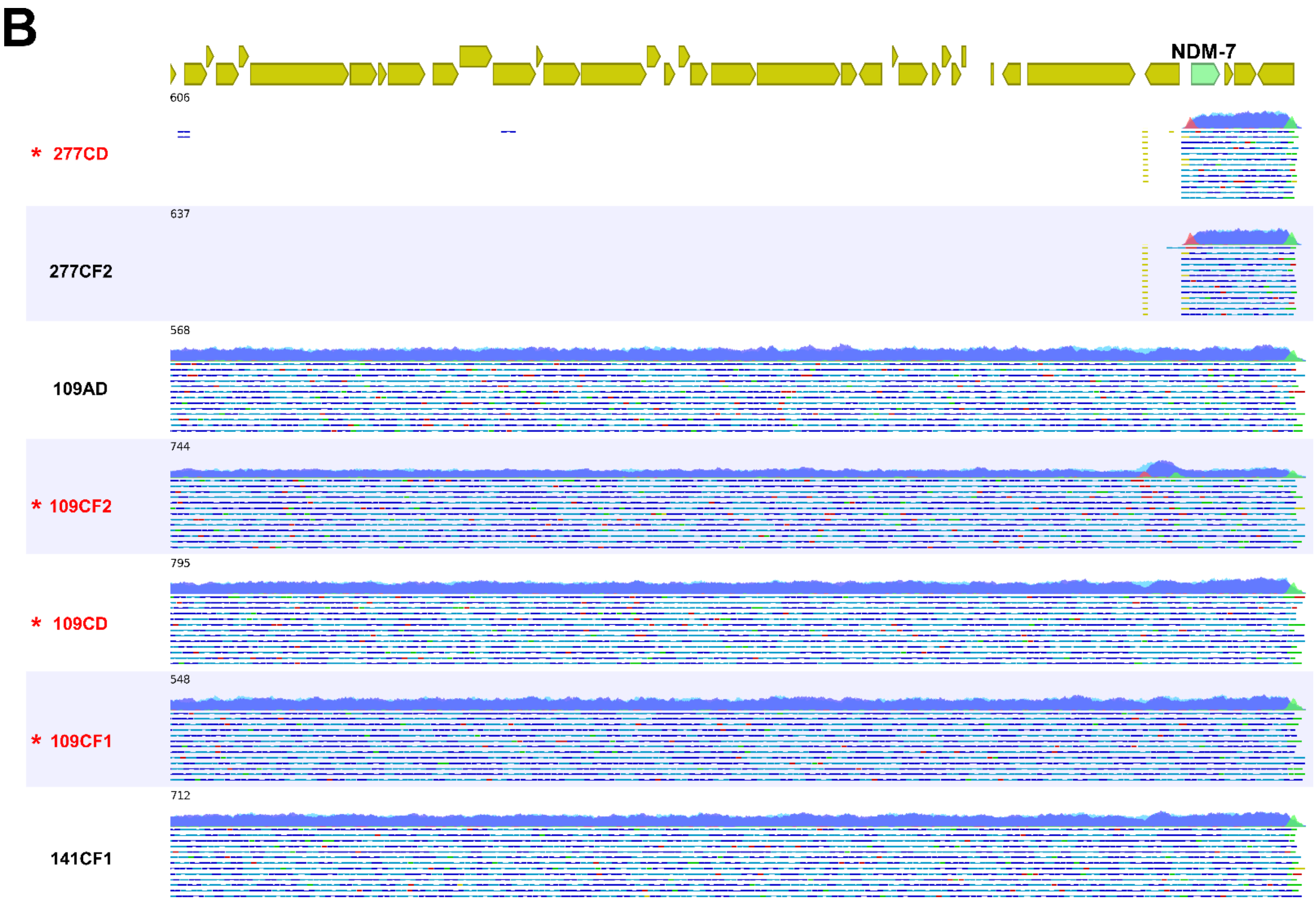

Supplementary Figure 3B: **Genes flanking *bla*$_{\mathrm{NDM\text{-}7}}$.** To access similarity of flanking regions, all *bla*$_{\mathrm{NDM\text{-}7}}$-positive isolates, for which FIA allele 1 and 6 were found, were assessed using a read mapping-based approach. Read files of respective isolates were mapped against the respective plasmid contig of #109AD. Each colored box represents a cluster (derived from core genome phylogeny). Copy strains are marked by red asterisks. Results indicate that flanking regions from newborn 109 (#109AD, #109CF1, #109CF2 and #109CD) differ from those of newborn 277 (#277CF2 and #277CD). However, similar flanking regions were found for newborn 109 and #141CF1 highlighting a potential plasmid transfer.

3 Supplementary tables

Supplementary table 2: MDRO- and CRO clusters identified by phylogenetic analysis.

transmission cluster	species	number of isolates	ST	resistance phenotype	carbape-nemases	ESBL genes
cluster I	*K. pneumoniae*	16	39	MDRO	-	CTX-M-15, SHV-187
cluster II	*K. pneumoniae*	9	39	MDRO	-	CTX-M-15, SHV-187
cluster III	*K. pneumoniae*	4	35	MDRO	-	CTX-M-15
cluster IV	*K. pneumoniae*	3	323	CRO	NDM-1	CTX-M-15, SHV-187
cluster V	*K. pneumoniae*	2	1427	MDRO	-	CTX-M-15, SHV-187
cluster VI	*K. pneumoniae*	2	17	MDRO	-	CTX-M-15, SHV-187
cluster VII	*K. pneumoniae*	5	17	MDRO	-	CTX-M-15, SHV-187
cluster VIII	*K. pneumoniae*	9	17	CRO	NDM-5	CTX-M-15, SHV-187
cluster IX	*K. pneumoniae*	5	45	MDRO*	-	CTX-M-15
cluster X	*K. pneumoniae*	3	-	MDRO	-	CTX-M-15, SHV-172
cluster XI	*K. pneumoniae*	5	48	MDRO	-	CTX-M-15
cluster XII	*K. pneumoniae*	2	268	MDRO	-	CTX-M-15
cluster XIII	*K. pneumoniae*	2	-	CRO	NDM-1	CTX-M-15, SHV-182
cluster XIV	*K. pneumoniae*	2	37	MDRO	-	CTX-M-3, SHV-187
cluster XV	*K. pneumoniae*	5	54	CRO	NDM-1	CTX-M-15, SHV-178
cluster XVI	*K. pneumoniae*	8	348	MDRO*	-	CTX-M-15, SHV-110
cluster XVII	*K. pneumoniae*	2	1801	MDRO	-	CTX-M-15, SHV-178
cluster XVIII	*K. pneumoniae*	3	14	MDRO	-	CTX-M-15, SHV-106
cluster XIX	*K. pneumoniae*	2	995	MDRO	-	CTX-M-15
cluster XX	*E. coli*	3	167	CRO	NDM-5	CTX-M-15

References

Andrews S, Lindenbaum P, Howard B, E. P. FastQC: a quality control tool for high throughput sequence data. (2010) Retrieved from: https://www.bioinformatics.babraham.ac.uk/projects/fastqc/ [accessed: 09.07.2019].

Charif, D., and Lobry, J. R. (2007). "SeqinR 1.0-2: A contributed package to the R project for statistical computing devoted to biological sequences retrieval and analysis," in (Springer, Berlin, Heidelberg), 207–232. doi: 10.1007/978-3-540-35306-5_10.

Jia, B., Raphenya, A. R., Alcock, B., Waglechner, N., Guo, P., Tsang, K. K., et al. (2017). CARD 2017: Expansion and model-centric curation of the comprehensive antibiotic resistance database. *Nucleic Acids Research* 45, D566–D573. doi: 10.1093/nar/gkw1004.

Jolley, K. A., and Maiden, M. C. J. (2010). BIGSdb: Scalable analysis of bacterial genome variation at the population level. *BMC Bioinformatics* 11, 595. doi: 10.1186/1471-2105-11-595.

Langmead, B., and Salzberg, S. L. (2012). Fast gapped-read alignment with Bowtie 2. *Nature Methods* 9, 357–359. doi: 10.1038/nmeth.1923.

Martin, M. (2011). Cutadapt removes adapter sequences from high-throughput sequencing reads. *EMBnet J* 17, 10. doi: 10.14806/ej.17.1.200.

Page, A. J., Cummins, C. A., Hunt, M., Wong, V. K., Reuter, S., Holden, M. T. G., et al. (2015). Roary: rapid large-scale prokaryote pan genome analysis. *Bioinformatics* 31, 3691–3693. doi: 10.1093/bioinformatics/btv421.

Price, M. N., Dehal, P. S., and Arkin, A. P. (2010). FastTree 2 - Approximately maximum-likelihood trees for large alignments. *PLoS ONE* 5, e9490. doi: 10.1371/journal.pone.0009490.

Prjibelski, A., Antipov, D., Meleshko, D., Lapidus, A., and Korobeynikov, A. (2020). Using SPAdes *de novo* assembler. *Current Protocols in Bioinformatics* 70. doi: 10.1002/cpbi.102.

R Core Team R: A language and environment for statistical computing. R Foundation for Statistical Computing, Vienna, Austria. (2018) Retrieved from: https://www.r-project.org/.

Seemann, T. Abricate. Retrieved from: https://github.com/tseemann/snp-dist [accessed: 24.06.2020].

Seemann, T. Mlst. Retrieved from: https://github.com/tseemann/mlst [accessed: 28.01.2020].

Seemann, T. Snp-dists. Retrieved from: https://github.com/tseemann/snp-dist [accessed: 28.04.2020].

Seemann, T. (2014). Prokka: rapid prokaryotic genome annotation. *Bioinformatics* 30, 2068–2069. doi: 10.1093/bioinformatics/btu153.

Wickham, H., Hester, J., and Francois, R. readr: Read rectangular text data. (2018) Retrieved from https://CRAN.R-project.org/package=readr.
